# GLP-1 receptor agonists modulate blood glucose levels in T2DM by affecting *Faecalibacterium prausnitzii* abundance in the intestine

**DOI:** 10.1097/MD.0000000000034978

**Published:** 2023-09-01

**Authors:** Lei Liang, Elizabeth Rao, Xuxiang Zhang, Bin Wu, Xiaoyun Su, Lin Chen, Rong Nie, Xin Nian

**Affiliations:** a The First Affiliated Hospital of Kunming Medical University, Department of Endocrinology, Kunming, China; b Tulane School of Medicine, New Orleans, LA.

**Keywords:** *Faecalibacterium prausnitzii*, GLP-1 receptor agonist, intestinal flora, *Lactobacillus delbrueckii*, type 2 diabetes

## Abstract

**Background:**

Glucagon-like peptide 1 (GLP-1) receptor agonists are a class of medications used to treat type 2 diabetes, including metformin, which is considered first-line therapy for type 2 diabetes. In recent years, GLP-1 receptor agonists (GLP-1 RAs) have been found to alter the composition and structure of gut flora and also promote the production of gut probiotics. However, there have been few clinical studies regarding the effects of GLP-1 RAs on gut flora. In this study, we investigated changes in the abundance of *Lactobacillus delbrueckii (L delbrueckii*) and *Faecalibacterium prausnitzii (F prausnitzii*) 1 week after administration of a GLP-1 RA in the clinical treatment of type 2 diabetes. The association with glycemic and body mass index (BMI) correlations was also explored.

**Methods:**

Twelve newly diagnosed patients with type 2 diabetes were examined for changes in the abundance of *L delbrueckii* and *F prausnitzii* by Fluorescence in Situ Hybridization 1 week after administration of GLP-1 RAs. Subjects BMI was measured and fasting glucose changes were detected using the glucose oxidase method, and *Spearman* correlation analysis was performed to explore their relevance.

**Results:**

There was no significant change in the abundance of *L delbrueckii* in the intestine (*P* = .695) and no significant correlation with BMI and fasting glucose levels (*R* = 0.134, *P* = .534) after the use of GLP-1 RA (R = −0.098, *P* = .647); *F prausnitzii* on the other hand had a significantly higher abundance (*P* = .002) and a significant negative correlation with fasting glucose level (R = −0.689, *P* < .001), but no significant correlation with BMI (R = −0.056, *P* = .796).

**Conclusion:**

*F prausnitzii* may be one of the pathways through which glucose is regulated in the treatment of type 2 diabetes by GLP-1 RAs.

## 1. Introduction

In recent years, the role of intestinal flora in the therapeutic mechanisms of type 2 diabetes mellitus (T2DM) has been of broad and current interest,^[1]^ and the involvement of intestinal flora in glucose-lowering effects in therapeutic regimens such as diet control, exercise therapy, weight loss surgery, and glucose-lowering drugs has been suggested.^[2]^ The mechanisms of intestinal flora in the treatment of T2DM have not been fully elucidated, but currently recognized mechanisms of occurrence include the short-chain fatty acid theory, the bile acid theory, and the endotoxin inflammation theory.^[3]^

Glucagon-like peptide 1 (GLP-1) receptor agonists are a class of medications known as themost widely used drugs for the treatment of T2DM because of their cardiovascular and metabolic benefits, such as improved cardiac capacity and significant weight loss.^[4]^ These medications include metformin, which is still regarded as first-line therapy for treating T2DM. GLP-1 receptor agonists (GLP-1 RA) are incretin-based drugs that act primarily in the human intestine to lower blood glucose by stimulating insulin secretion and lowering glucagon secretion.^[3]^ Kato et al^[5]^ found that after 16 hours of use of GLP-1 RAs, gut flora in patients was significantly altered at the phylum level, with a significant reduction in phylum *Bacteroidetes* and a trend towards an increase inphylum *Actinobacteria*. At the genus level, there was a significant decrease in the abundance of *Ruminococcus*. In addition, a recent study found significant differences in the beta diversity of gut flora between patients with good glycemic control using and those without using GLP-1 RAs, where the abundance of probiotics such as *Alfalfa spp*. and *Butyricoccus spp*. were positively correlated with glycemic reduction. These probiotics likely have a positive role in the treatment of T2DM with GLP-1 RAs.^[6]^ Currently, this is the only clinical study we are aware of that reports on GLP-1 RAs and their effect on the intestinal flora. Beyond that, we are not aware of any studies related to the use of GLP-1 RAs and probiotic abundances.

The research conducted in this paper is focused on the effect of GLP-1 RAs on 2 types of bacteria present in the intestinal flora, *Lactobacillus delbrueckii (L delbrueckii*) and *Faecalibacterium prausnitzii (F prausnitzii*). In current studies on the intestinal flora, *Lactobacillus spp*. has been regarded as one of the most vital probiotics, and the abundance of *Lactobacillus* is often a focus topic for probiotic research.^[7]^ *L delbrueckii*, as a representative species of *Lactobacillus spp.*, has significant probiotic properties, including anti-inflammatory effects in gastrointestinal tract disease treatment and pathogen inhibition.^[8]^ *L delbrueckii* is a gram-positive rod-shaped homofermentive lactic acid bacteria that is involved in the production of dairy products such as cheese and yogurt and produces acetaldehyde, the main flavor component in yogurt.^[9]^ On the other hand *F prausnitzii*, as the only species currently identifiedin the *Faecalibacterium* genus in the gut, is considered a crucial probiotic for measuring the health of the intestinal flora.^[10]^ *F prausnitzii* is a butyrate-producing bacterium that has been found at about 5% to 10% in abundance in the intestinal flora of healthy adults, and is one of the most abundant species in the known intestinal flora.^[11]^

In this study, Fluorescence in Situ Hybridization (FISH) was used to observe changes in blood glucose, body mass index (BMI), and abundance of *L delbrueckii and F prausnitzii* in the guts of human adult patients before and after the use of GLP-1 RAs to analyze the effect of GLP-1 RAs on these 2 bacteria in the intestinal flora. This study also aims to clarify whether changes in blood glucose and BMI following treatment with GLP-1 RAs for T2DM are related to the abundance of *F prausnitzii* and *L delbrueckii* in the human gut.

## 2. Materials and methods

### 2.1. Participants

We recruited subjects that were newly diagnosed with T2DM at the outpatient clinic of the Department of Endocrinology of the First Affiliated Hospital of Kunming Medical University. They were enrolled in the study by signing an informed consent form for a 1 week trial after meeting inclusion and exclusion criteria. The study was conducted in strict accordance with the Declaration of Helsinki and the 4 Principles of Ethics, and all subjects provided informed consent. The study was registered with the Chinese Clinical Trials Registry (registration number: ChiCTR2200063198) and ethical review approval was obtained from the First Affiliated Hospital of Kunming Medical University (approval number: 2022L79).

Inclusion criteria: patients with a clinically new diagnosis of T2DM; agreement to participate in this study and signed an informed consent form after being informed of the study.

Exclusion criteria: acute and chronic diseases of the gastrointestinal, cardiovascular, and nervous systems that may affect gut flora; intake of antibiotics, glucocorticoids, and other medications that may affect gut flora within the last 3 months; women who are nursing or pregnant; prior long history of smoking and alcohol consumption.

### 2.2. Stool collection

Before the start of the test, subjects were asked to collect 2g of fasting stool in a sterile tube in the early morning. The samples were immediately sent to the laboratory for cell morphological fixation. Subjects were then administered with GLP-1 RAs for a duration of 1 week under observation and monitoring of the subjects general condition. Meals were provided uniformly to all subjects during the trial and were guaranteed to be consistent by the hospital’s nutritional dining room, in order to reduce the effect of diet on the trial. After 1 week of GLP-1 agonist administration, early morning fasting stools were again collected from the subjects and sent to the laboratory for cell fixation.

### 2.3. Blood collection

Immediately following collection of patients’ stool samples in the early morning, 10 mL of fasting venous blood was drawn from the subject in a sterile tube and sent to the laboratory for storage at −80ºC in the refrigerator both before and after administration of GLP-1 RAs for 1 week.

### 2.4. Measurement of BMI

BMI = Weight/ Height^2^ (kg/m^2^) was measured by the researcher after the subjects removed their shoes, hats and coats both before and after administration of GLP-1 RAs for 1 week.

### 2.5. Stool sample pretreatment

The collected stool samples needed to be sent to the laboratory within 1 hour for preprocessing to prevent bacterial death due to environmental factors and to limit changes in cell morphology. The samples were fixed with 4% Paraformaldehyde at 4°C for 3 hours, then centrifuged. The sediment was rinsed 3 times with sterile water, then suspended in 50% alcohol and stored at −20°C.

### 2.6. Fluorescence in situ hybridization

Specific oligonucleotide probes Lbd (5’- AAG GAT AGC ATG TCT GCA -3’) and FPR-1 (5’- GTC GAA CGA GCG AGA GAG GAG CTT GCT TTC TCA AGC GAG T -3’) labeled with fluorochrome CY3 were selected and FISH was performed according to the procedure described by Kong et al.^[12]^ Samples were mixed with CY3 probes in 0% formamide hybridization solution for 3 hours at 46°C and protected from light, followed by elution of the hybridization solution in eluent and staining of total bacteria of the samples using 4’,6-diamino-2-phenylindole anti (DAPI, Beijing Lanjike Technology Co., Ltd.).

### 2.7. Microscopic observation and cell counting

Each sample was observed microscopically using a fluorescence microscopic imaging analysis system DP72 (Olympus Corporation, Japan) and photographed using celsens software. Images of Cy3 and DAPI staining in the same field of view were taken as 1 group. To reduce errors, at least 10 sets of images for each sample were taken. Cell counts were performed for each set of images using ImageJ software. Bacterial abundance per group was calculated as the number of Cy3-stained cells/number of DAPI-stained cells, and the final bacterial abundance for each sample was taken as the median of the 10 groups. The 2 researchers counted the bacteria in the samples, averaged them when they were close to each other, and recalculated them when they were much different.

### 2.8. Measurement of blood glucose

The blood glucose content of the subjects was measured using a glucose oxidase assay kit (Shanghai Bioengineering Co., Ltd.).

### 2.9. Statistical analysis

The data was processed using SPSS 19.0 statistical software (IBM Corp, Armonk, NY). Measurement data conforming to normal distribution were expressed as (X ± S), and the *t test* was used to compare 2 groups; the measurement data with non-normal distribution were expressed as M (P25, P75), and the *Wilcoxon test* was used to compare 2 groups; correlation analysis was performed by Spearman coefficient test, and *P* < .05 was considered a statistically significant difference.

## 3. Results

### 3.1. Subject information

**Figure 1.**
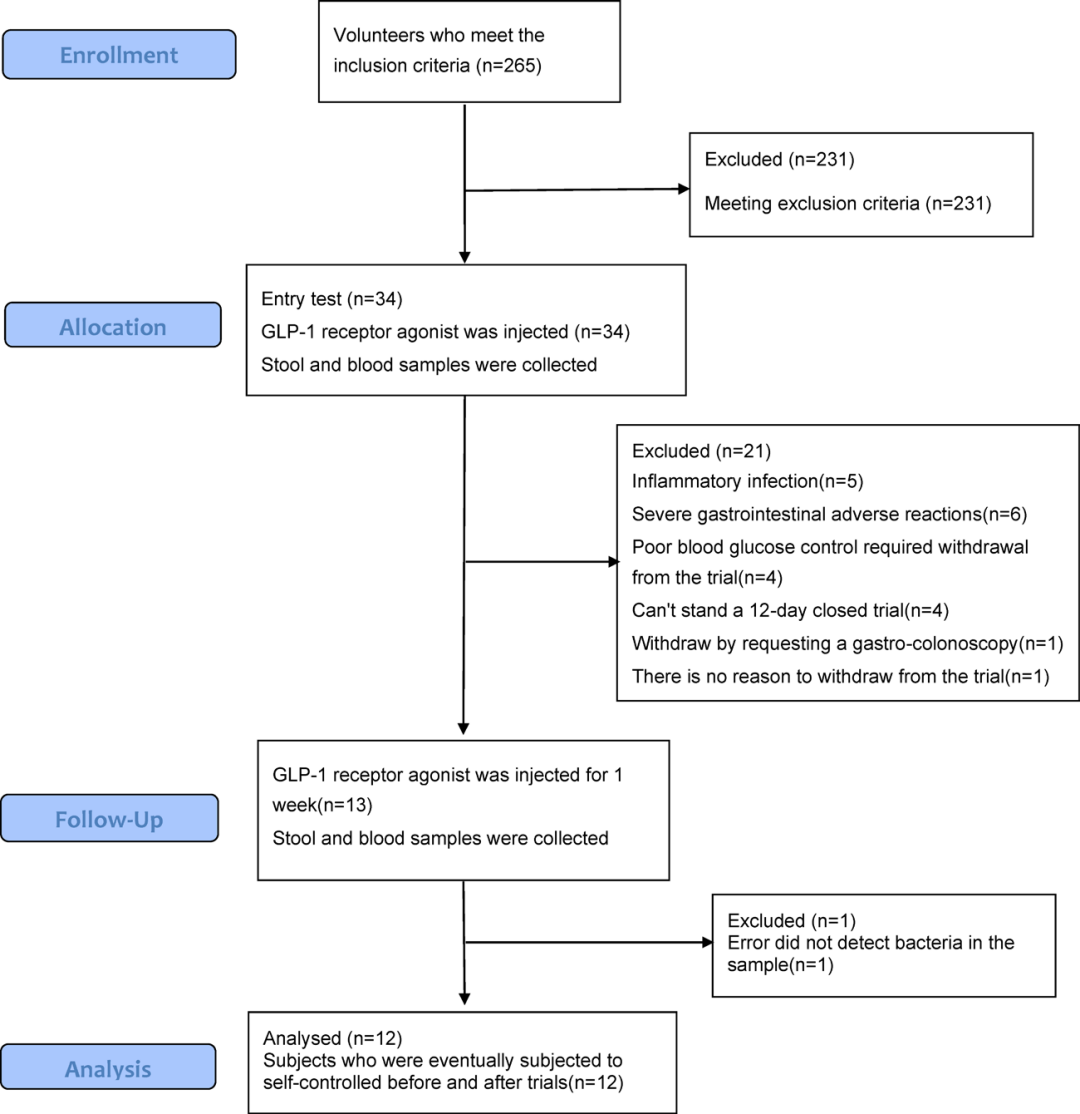
Test flow chart.

When analyzing results from the second stool sample from subjects following GLP-1 RA administration for 1 week, it should be noted that some of the subjects had taken antibiotics or other medications that might affect the intestinal flora during the study period because subjects were already hospitalized and needed to complete relevant tests. Some subjects were diagnosed with inflammation, and acute or chronic diseases. Some subjects even underwent gastroscopy during the study period, which may have affected the outcome of the gut flora, as a result, these subjects had to be terminated (Fig. 1). Following these terminations, a total of twelve subjects were ultimately included in the study, consisting of 6 males and 6 females, with an age range of 54.25 ± 13.18.

### 3.2. Changes in the abundance of *L delbrueckii* and *F prausnitzii* before and after the use of GLP-1 RAs

**Figure 2.**
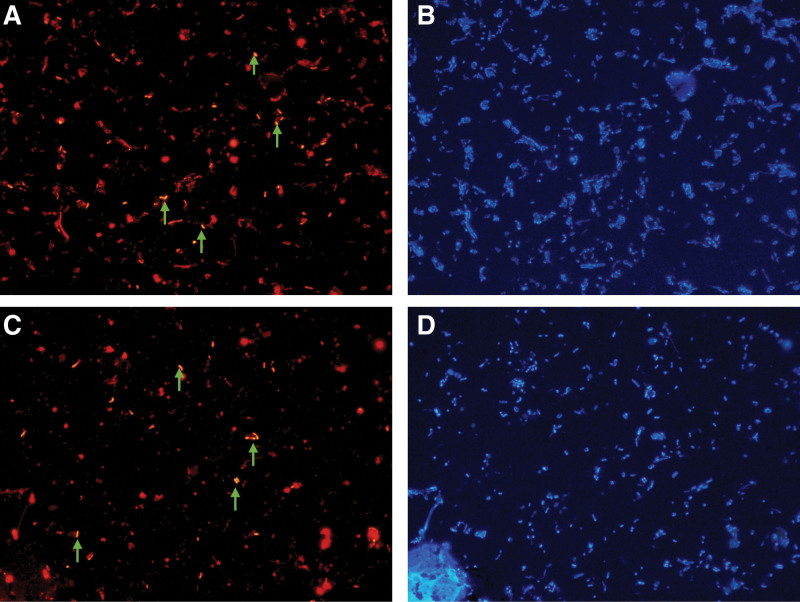
*Lactobacillus delbrueckii* abundance changes before and after administration of GLP-1 receptor agonist, green arrows are Lbd probes for *Lactobacillus delbrueckii*. (A) Dyed by the probe before GLP-1 RA and (B) dyed by the corresponding DAPI; (C) dyed by the probe after 1 week of GLP-1 RA and (D) dyed by the corresponding DAPI. GLP-1 = glucagon-like peptide 1.

**Figure 3.**
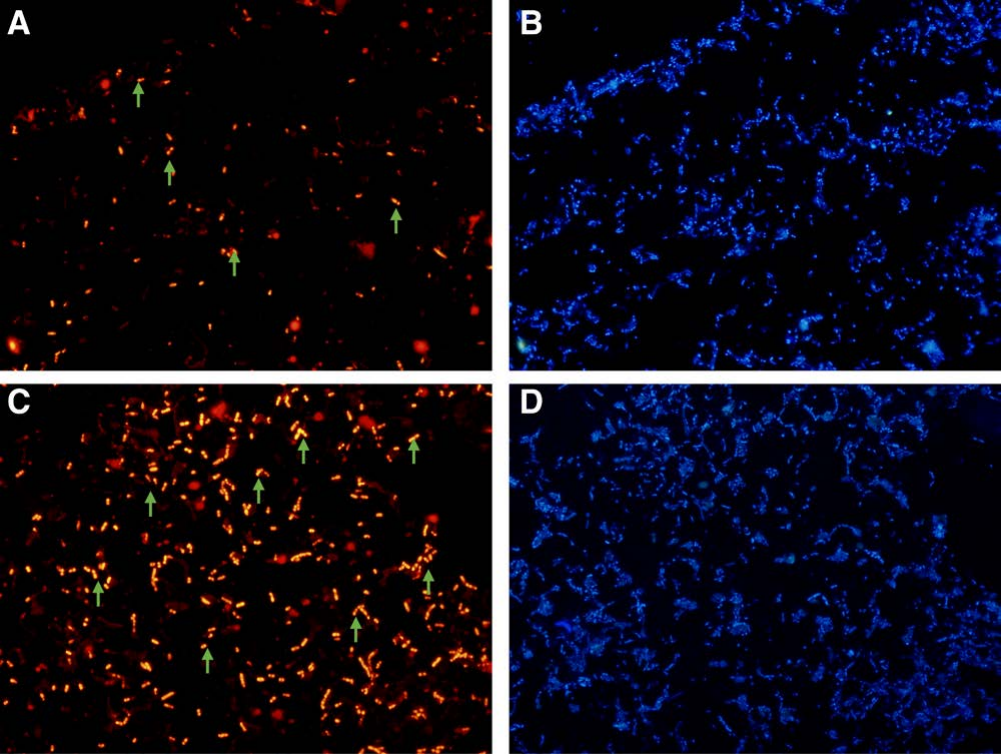
*Faecalibacterium prausnitzii* abundance changes before and after administration of GLP-1 receptor agonist, green arrows are FPR-1 probes for *Faecalibacterium prausnitzii*. (A) Dyed by the probe before GLP-1 RA and (B) dyed by the corresponding DAPI; (C) dyed by the probe after 1 week of GLP-1 RA and (D) dyed by the corresponding DAPI.GLP-1 = glucagon-like peptide 1.

We observed the changes in the abundance of *L delbrueckii* and *F prausnitzii* before and after the use of GLP-1 RA by using the FISH technique (Figs. 2 and 3).

Figure 2A shows *L delbrueckii* labeled with Lbd probe before the use of GLP-1 RA, and Figure 2B shows all bacteria corresponding to Figure 2A labeled with DAPI staining. Figure 2C shows *L delbrueckii* labeled with the Lbd probe after the use of the GLP-1 RA, and Figure 2D shows all bacteria corresponding to Figure 2C labeled with the DAPI stain.

Figure 3A shows *F prausnitzii* labeled with the FPR-1 probe before the use of GLP-1 RAs; Figure 3B shows all bacteria tagged with the DAPI stain in the same field of view corresponding to Figure 3A. Figure 3C shows *F prausnitzii* labeled with the FPR-1 probe after application of the GLP-1 RA, and Figure 3D shows all bacteria labeled with DAPI staining in the same field of view corresponding to Figure 3C for all bacteria.

**Figure 4.**
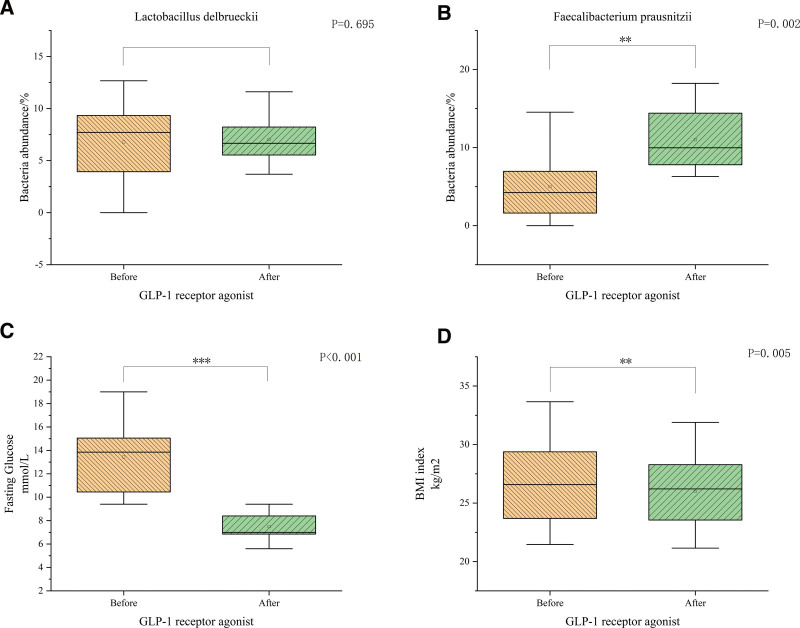
(A) Is a box plot of the abundance of *Lactobacillus delbrueckii* before and after use of GLP-1 receptor agonists, (B) is a box plot of the abundance of *Faecalibacterium prausnitzii* before and after the use of GLP-1 receptor agonists, (C) is a box plot of fasting glucose changes before and after the use of GLP-1 receptor agonists, and (D) is a box plot of BMI before and after the use of GLP-1 receptor agonists.*:*P* < .05;**:*P* < .01;***:*P* < .001. BMI = body mass index, GLP-1 = glucagon-like peptide 1.

We found that *L delbrueckii* abundance was 7.71 (3.74, 9.39) before the use of GLP-1 RA and 6.67 (5.43, 8.43) after the use of GLP-1 RA The *Wilcoxon* rank sum test analysis showed no statistically significant difference in bacterial abundance before and after GLP-1 RA administration in *L delbrueckii (P* = .695) (Fig. 4A). On the other hand *F prausnitzii* abundance was 4.24 (0.81, 6.97) before and 9.98 (7.65, 14.88) after the use of GLP-1 RA. The *Wilcoxon* rank sum test analysis showed a significant difference in bacterial abundance before and after administration of GLP-1 RA in *F prausnitzii (P* = .002) (Fig. 4B).

### 3.3. Changes in blood glucose and BMI index

We found that patient’s fasting blood glucose significantly decreased after 1 week of GLP-1 RA administration (*P* < .001) (Fig. 4C); in addition, patients BMI also significantly decreased after 1 week of GLP-1 RA administration (*P* = .005) (Fig. 4D).

### 3.4. Correlation analysis

**Figure 5.**
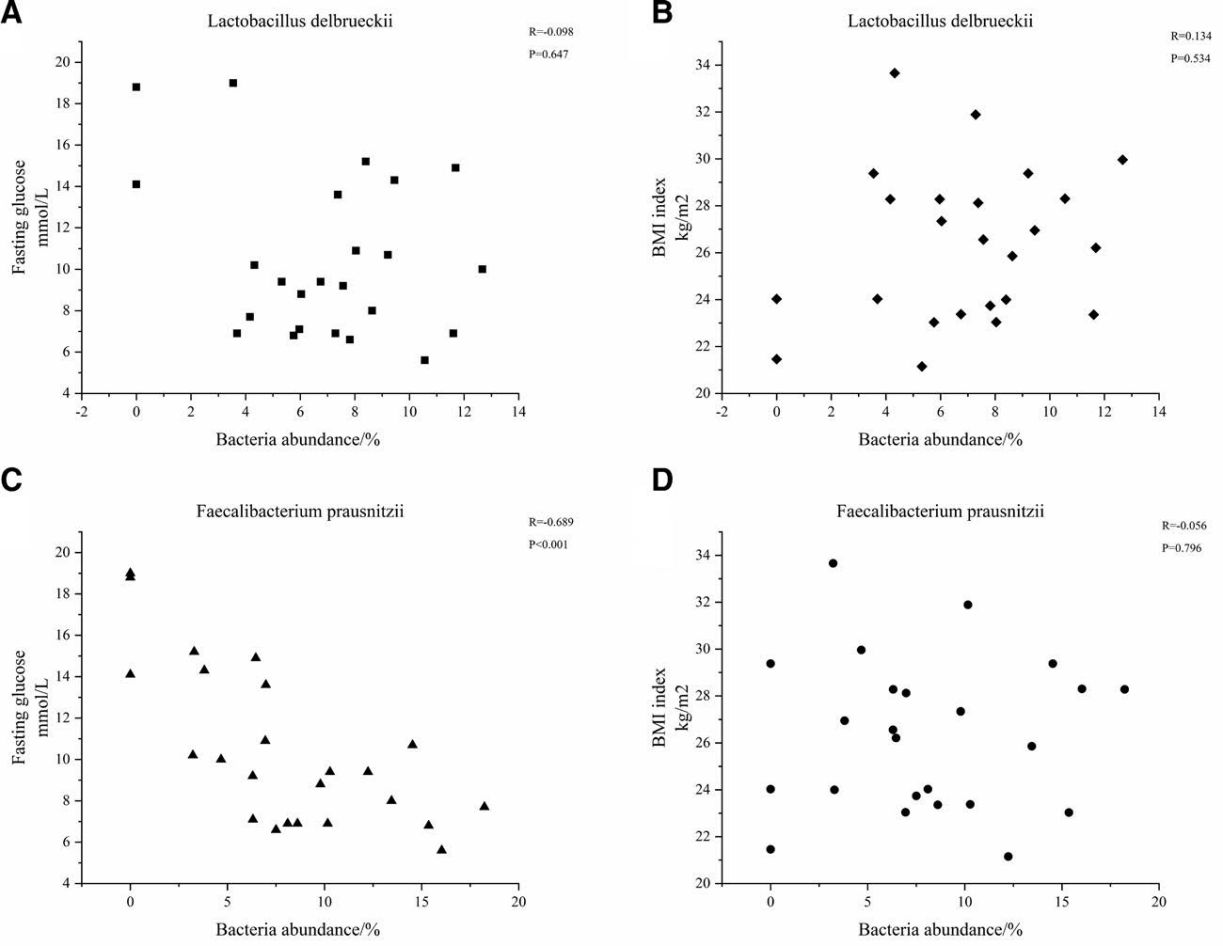
(A) Is the scatter plot of the correlation analysis between *Lactobacillus delbrueckii* and fasting glucose, (B) is the scatter plot of the correlation analysis between *Lactobacillus delbrueckii* and BMI, (C) is the scatter plot of the analysis of the correlation between *Faecalibacterium prausnitzii* and fasting glucose, and (D) is the scatter plot of the analysis of the correlation between *Faecalibacterium prausnitzii* and BMI. BMI = body mass index.

We used Spearman correlation analysis to explore whether the abundance of *L delbrueckii* and *F prausnitzii* in the gut is correlated with blood glucose levels and BMI when using GLP-1 RAs to treat T2DM. The results showed that fasting blood glucose was not significantly correlated with the abundance of *L delbrueckii* in the gut (R = −0.098, *P* = .647) (Fig. 5A); however, it was negatively correlated with the abundance of *F prausnitzii* (R = −0.689, *P* < .001) (Fig. 5C). In contrast, there was no significant correlation (*R* = 0.134, *P* = .534) between BMI and both *L delbrueckii* abundance (Fig. 5B) and *F prausnitzii* abundance (Fig. 5D) in the gut following administration of GLP-1 RAs (R = −0.056, *P* = .796).

## 4. Discussion

FISH is a molecular biology technique often used for the identification and enumeration of intestinal microorganisms directly under a fluorescent microscope by hybridization of fluorescent dye-impregnated oligonucleotide probes with cells that have been pretreated in advance.^[13]^ In the past, it has mostly been used for the study of environmental microorganisms and gut microbes^[14,15]^; in recent years, the usage of FISH has gradually expanded and can be applied to the screening of tumor cells in clinical settings^[16]^ and the examination of genetic chromosomes.^[17]^ FISH allows for the design of oligonucleotide probes specific for different bacteria with higher specificity than the primers used in 16 seconds rRNA and macroeconomic PCR amplification of target fragments. It also has a higher accuracy in detecting the abundance of individual bacteria.^[18,19]^

In this study, stool samples from subjects recently diagnosed with T2DM were examined by FISH before and after a 1 week duration of GLP-1 agonist administration. *F prausnitzii* was found to be significantly more abundant in the human gut after the use of GLP-1 RAs, while *L delbrueckii* showed no significant differences before and after GLP-1 RA administration. We also validated the glucose-lowering and weight loss capabilities of GLP-1 RAs. We found that following usage of a GLP-1 RA, a patient’s fasting glucose can drop to ideal levels. Regarding weight loss, patients who were administered GLP-1 RAs had an average BMI reduction of 0.64 kg/m^2^, which was statistically significant.

*F prausnitzii* is a butyrate-producing bacterium with a well-known anti-inflammatory potential in the host.^[20]^ Butyrate provides about 10% of the host’s daily energy requirements, protects against pathogenic invasion, modulates the immune system, and inhibits the progression of autoimmune diabetes.^[21]^ Moderate levels of butyrate can also contribute to the treatment of T2DM by improving high-fat diet-induced insulin resistance through epigenetic regulation and mitochondrial β-oxidation.^[22]^ Studies have pointed out that high levels of butyric acid can stimulate GLP-1 secretion, enhance insulin sensitivity through the cAMP signaling pathway, and inhibit gastric emptying in humans.^[23]^This mechanism of action is similar to that of GLP-1 RAs.

Moreover, *F prausnitzii* can promote bile acid levels in vivo,^[24]^ maintain intestinal barrier function and improve intestinal inflammatory status by regulating energy metabolism.^[25]^ Bile acids can also regulate the secretion of intestinal hormones such as GLP-1 and PYY in the body, acting in the same way as short-chain fatty acids such as butyrate, which in turn affect appetite and energy intake and directly regulate blood glucose levels in diabetic patients.^[26]^

In addition to improving the intestinal inflammatory response in diabetes through indirect factors such as short-chain fatty acids and bile acids, *F prausnitzii* can also directly secrete microbial anti-inflammatory molecules to stabilize intestinal epithelial cell permeability and restore intestinal barrier structure and function in diabetic conditions by regulating intestinal epithelial tight junction pathways and ZO-1 expression.^[18]^ Arguably, *F prausnitzii* affects the treatment of T2DM through 3 main mechanisms of action: the short-chain fatty acid theory, the bile acid theory, and the endotoxin inflammation theory.

One study found that *F prausnitzii* abundance in the intestine of diabetic and obese patients was significantly decreased and negatively correlated with glycated hemoglobin HbA1c values.^[27]^ In contrast, in our study, we found a significant negative correlation between fasting glucose and *F.prausnitzii* abundance in the gut of patients treated with GLP-1 RAs, but no significant correlation between BMI and *F prausnitzii* abundance. We suggest that while *F prausnitzii* may be one of the pathways of glucose regulation in the treatment of T2DM with GLP-1 RAs, the weight loss effects of GLP-1 RAs are not directly related to *F prausnitzii*.

Notably, a recent study found that *L delbrueckii* improved fasting glucose, fetuin-a, and Sestrin-3 levels in type 2 diabetic mice.^[28]^ Fetuin-a and Sestrin-3, 2 circulating proteins in the organism that play a vital role in the process of insulin resistance,^[29,30]^ are good targets for the regulation of insulin sensitivity.^[28]^ In addition, another META analysis also indicated that *L delbrueckii* could reduce fasting glucose, glycated hemoglobin, and insulin levels in patients with T2DM.^[31]^ However, our study found no significant change in *L delbrueckii* abundance after treatment with GLP-1 RAs and no significant correlation with fasting glucose levels and BMI. This suggests that *L delbrueckii* is not involved in the hypoglycemic and weight loss effects of GLP-1 RAs used in the treatment of T2DM, and that GLP-1 RAs have no direct effect on *L delbrueckii* abundance in the gut.

As there are few clinical studies of gut flora, this study is the first in which we explore the relationship between the hypoglycemic effects of GLP-1 RAs and gut flora. Due to the many factors affecting gut flora, strict control of inclusion and exclusion criteria would result in insufficient sample sizes, making it difficult to collect valid sample sizes. Later, due to the liberalization of the control of the novel coronavirus epidemic, most people were infected with the virus. To prevent the effects of novel coronavirus on the gut flora, we ultimately had no choice but to terminate the trial, with only 12 samples meeting the criteria to be included in the final study.

Since this study was an initial exploratory study, despite the small sample size, our results suggest that the variability in the abundance of *F prausnitzii* with the use of GLP-1 receptor agonists is significant. We believe this is sufficient to provide a theoretical basis for our later in-depth exploration of the mechanism of action of the gut flora in GLP-1 RAs.

## 5. Conclusion

*F prausnitzii* is considered a marker of intestinal health, and its abundance has been strongly associated with the development of T2DM and obesity. In this study, we examined the changes in intestinal flora in patients with T2DM before and after the use of GLP-1 RA by FISH and found that the abundance of *F prausnitzii* in the intestine increased significantly after 1 week of treatment with GLP-1 RA for T2DM. *F prausnitzii* also showed a significant negative correlation with fasting glucose of patients, which suggests that *F prausnitzii* may be one of the pathways through which GLP-1 RAs regulate blood glucose.

## Author contributions

**Conceptualization:** Lei Liang, Xiaoyun Su.

**Data curation:** Elizabeth Rao.

**Formal analysis:** Lin Chen, Rong Nie.

**Funding acquisition:** Xin Nian.

**Methodology:** Lei Liang, Xin Nian.

**Project administration:** Xuxiang Zhang, Xin Nian.

**Resources:** Xin Nian.

**Software:** Elizabeth Rao, Xuxiang Zhang, Bin Wu, Xin Nian.

**Supervision:** Bin Wu, Xin Nian.

**Writing – original draft:** Lei Liang.

**Writing – review & editing:** Lei Liang.
